# Enhancing sand screen performance with integrated slurry testing and CFD-DEM modelling

**DOI:** 10.1016/j.heliyon.2024.e40877

**Published:** 2024-12-16

**Authors:** Noorhaslin Che Su, Sofyah Anis Izwani Jusof, Aimi Zahraa Zainal, Yeong Yuan Jian, Chan Kai Xian, Afiq Mohd Laziz, Muhmmad Fadhli Muhammad, Javed Akhbar Khan, Abdul Hazim Abdullah, Mohd Azuwan Maoinser

**Affiliations:** a Institute of Sustainable Energy Resources, Universiti Teknologi PETRONAS, Bandar Seri Iskandar, Perak, 32610, Malaysia; b Department of Mechanical Engineering, Universiti Teknologi PETRONAS, Bandar Seri Iskandar, Perak, 32610, Malaysia; c Department of Petroleum Engineering, Universiti Teknologi PETRONAS, Bandar Seri Iskandar, Perak, 32610, Malaysia; d Department of Chemical Engineering, Universiti Teknologi PETRONAS, Bandar Seri Iskandar, Perak, 32610, Malaysia; e PETRONAS Research Sdn. Bhd., Petronas Research & Scientific, Government and Private Training Centre Area, 43000 Bandar Baru Bangi, Selangor, Malaysia; f College of Electrical & Mechanical Engineering, China University of Petroleum, Qingdao, 266580, China

**Keywords:** Particle size distribution, Slurry test, Constant flow rate, Sand retention test, Wire wrapped, CFD-DEM, Screen permeability

## Abstract

Understanding the behavior of sand screens is crucial for optimizing sand control strategies and preventing wellbore failure, which can significantly impact reservoir management and production efficiency. This paper presents a comprehensive experimental and numerical modeling study on sand screen performance, aimed at providing insights prior to real-field applications. The study evaluated a 200-μm wire-wrapped screen (WWS) using slurry tests to determine the amount of sand retained, sand produced and retained permeability to assess screen efficiency. The particle size distribution (PSD) of sand samples was crucial for selecting the appropriate screen size, with results showing that highly uniform and well-sorted sand exhibited higher sand retention and lower sand production due to reduced fines content and maximized void spaces, enhancing fluid flow while mitigating sand production. A coupled Computational Fluid Dynamics-Discrete Element Method (CFD-DEM) simulation platform was employed to model particle-fluid interactions, using an optimal mesh size of 0.005 m. The simulation results showed a high degree of correlation with experimental data, with 93.1 % similarity in sand retention and 63.22 % in sand production. These findings underscore the potential of CFD-DEM simulations in accurately replicating complex sand control processes and optimizing screen performance for enhanced wellbore stability and production efficiency.

## 1 Introduction

In petroleum sector, extracting oil and gas from unconsolidated reservoirs typically results in sand production. Over the past twenty years, significant expenditures have been allocated to combat pipeline blockage, equipment erosion and decreased oil production resulting from sand-related issues [[1], [2], [3], [4], [5]]. This issue has sparked extensive discussions among researchers. Therefore, to overcome this challenge, numerous methods for sand control have been developed. Key considerations in selecting a suitable sand control method include cost-effectiveness, efficiency in retaining different types of sand, and durability [6]. These factors are crucial for optimizing well performance and longevity.

Various technologies for implementing sand control ranging from mechanical methods such as gravel packing [[7], [8], [9]] and sand screens [[10], [11], [12], [13]] to chemical treatments [[14], [15], [16]] and integrated devices such as autonomous inflow control devices (AICDs) [17]. Gravel packing, a commonly referenced method, involves placing gravel around the well screen to filter sand particles. However, sand-rich sedimentary facies, such as mouth bars and overbanks, often merge to form compound sand bodies, presenting unique challenges [18]. Fine particle content in these formations can cause clogging of gravel packs, impacting their efficiency [19]. Chemical treatments involve the use of chemicals to consolidate or alter the formation to prevent sand production. Injecting chemicals into the formation to mitigate sand production poses specific challenges, including high costs, pumping issues, and a significant decrease in permeability due to the filling of the porous media around the wellhead [20,21]. Furthermore, contamination of groundwater and surrounding environments might occur if not handled properly. AICDs are sophisticated devices that require precise engineering and maintenance. Their complexity can lead to higher installation and maintenance costs compared to simpler sand control methods. Among the various sand control methods, sand screens stand out for their overall benefits in well production operations. In addition to its efficiency in mitigating sand, the sand screen demonstrates cost-effective installation, excellent mechanical robustness, simple design and ease of installation [22,23]. Thus, a sand screen is often preferred for sand control in unconsolidated reservoirs.

Sand screens installed in production wells play a vital role in mitigating sand production by serving as downhole filters, preventing sand passage while enabling the flow of hydrocarbons [24,25]. The selection of the screen is influenced by the characteristics of sand formation in the targeted well [26]. Inappropriately tailored designs for specific reservoir conditions often result in screen failures, such as plugging, bursting, and collapsing. To date, various screen technologies have revolutionized the industry, including slotted liners [[27], [28], [29]], wire-wrapped [[30], [31], [32]], and premium mesh screens [[33], [34], [35]]. Each design has its own unique construction features. Consequently, selecting the most appropriate sand screens is essential to ensure a good hydrocarbon productivity.

In order to investigate the particle interactions between the fluid with the sand screen and to understand the fluid flow mechanism, a slurry test, also known as the constant flow rate test, was conducted. The main objective of the slurry test is to analyze the behaviour of a fluid mixture (slurry) in the presence of sand as it is injected through the system. This laboratory-based sand retention test is designed to evaluate the screen performance before real field applications [27,36]. In a previous study, this test was used to simulate the erosional failure of the formation on the screen that occurs with the open hole region between the screen and the reservoir sand. However, the conventional slurry test has significant limitations in providing detailed insights into particulate-fluid interactions during testing. The experimental data are unable to adequately capture dynamic behaviours and mechanisms associated with sand screen retention which can lead to inaccuracies in assessing the effectiveness of the screen. Therefore, to address these shortcomings, it is essential to incorporate simulation methods that can replicate experimental conditions and predict the retention mechanism involved in the process.

This study aims to evaluate the performance of a wire-wrapped screen (WWS) through slurry testing using sand extracted from offshore fields in the Gulf of Thailand. Controlling accuracy and mimicking real field conditions require studying the sand particle size distribution as an initial step for sand retention tests [[36], [37], [38], [39]]. A prior analysis of the size and properties of the sand samples was conducted. The results provide the amount of solid produced through the screen, the amount of sand retained on the screen, and the retained permeability of the screen over time as formation is deposited onto the screen. Complementing the experimental work, the Computational Fluid Dynamics (CFD) and discrete element method (DEM) simulations are performed to investigate the dynamics of the fluid and the interaction of sand particles in the slurry test respectively. The coupled analysis of CFD and DEM allowed more accurate predictions of overall sand retention behavior. Geometry development is a critical step in ensuring the validity and accuracy of the simulations. It offers a theoretical base for a general model of the interaction between particles, as well as with the surrounding fluid and the screen. In recent years, the CFD-DEM approach has been widely accepted as an effective tool for studying various particle-fluid systems as reviewed by different researchers [22,[40], [41], [42]]. Previously, Wu and colleagues [43] successfully developed a numerical model for an enhanced sand retention test (SRT). However, although extensive research had been done on the numerical modelling of SRT through the CFD-DEM approach, none of the papers used actual sand screen geometry. Simplified geometries, often reduced to rectangular designs, were commonly employed. This study has successfully proposed a realistic model that closely mirrors the actual design of the screen and slurry setup. This ensures a more accurate simulation of fluid dynamics and particle interactions. The proposed model has also been validated with an experimental design, which confirms its effectiveness. By maintaining the integrity of the real system, we enhance the reliability and accuracy of the sand retention test results, offering a significant improvement over previous simplified design. This novel approach strengthens the general understanding and assessment of sand control mechanisms in real-world applications.

## 2 Methodology

### 2.1 Formation characterization

**Fig. 1:**
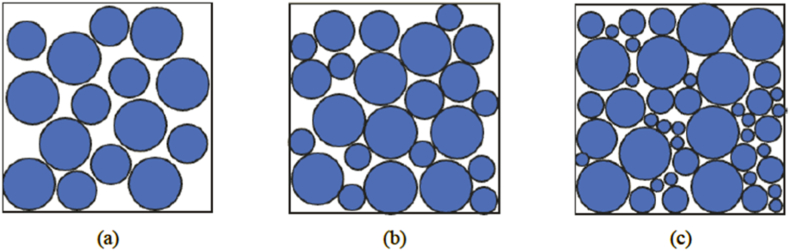
Illustration of sand samples with (a) uniform and well sorted (b) non-uniform and poorly sorted (c) non-uniform, poorly sorted and high percentage of fines.

Formation sand characterizations are crucial parameters for studying the performance of the screen. Thus, particle size distribution study (PSD) is used to analyze the size and properties of the sand samples. Laser Particle Size Analyzer (LPSA) is one of the established techniques for measuring the sand size. To effectively visualize this data, semilogarithmic curves were plotted based on the result collected. This approach offers a comprehensive understanding of sand's characteristics which is essential for designing optimal sand control in the targeted field. The sand characteristics can be classified based on the Uniformity Coefficient (UC), Sorting Coefficient (SC) and percentage of fines. The calculation for UC, SC and percentage of fines was shown in Eqs. (1), (2), (3) [38] and the classification criteria are shown in Table 1. The illustration of sand sample characteristics is shown in Fig. 1.

(1) Uniformity coefficient, UC = D40/D90

(2) Sorting coefficient, SC = D10/D95

(3) Percentage of fines = Sum of particles less than 45 μm

**Table 1:** The type of coefficient and classification of sand samples [10].

Type of Coefficient	Coefficient Classification	
**Uniformity Coefficient, UC**	UC < 3	Uniform
	3< UC < 5	Non-Uniform
	UC > 5	Highly non-uniform
**Sorting Coefficient, SC**	SC < 10	Well-sorted
	SC > 10	Poorly-sorted

### 2.2 Screen selection

**Fig. 2:**
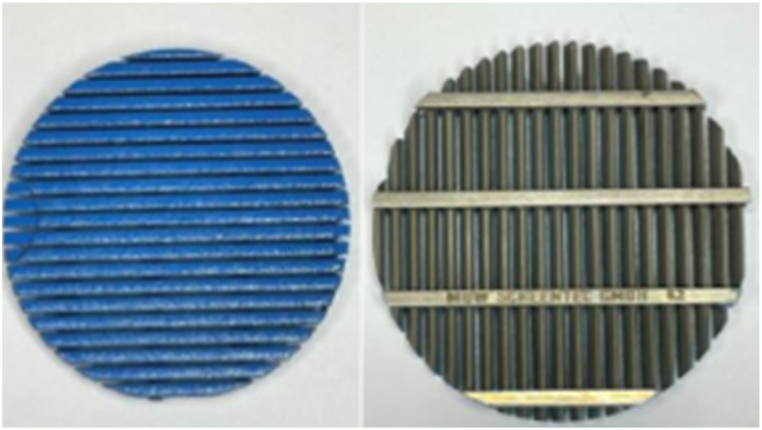
The wire-wrapped screen with a 200-μm opening.

The wire-wrapped screen criteria used in this study are shown in Fig. 2 and Table 2 respectively.

**Table 2:** Wire-wrapped screen criteria.

Screen Opening Size (micron)	Screen Area (cm^2^)	Permeability (mD)
200	45.6	11,436.95

### 2.3 Slurry test

#### 2.3.1 Preparation of slurry solution

A slurry solution was prepared by mixing sand samples with 20 ml of a solution containing polymer and glycerin. The mixture is vigorously stirred to achieve homogeneity and prevent sedimentation of the sand particles, resulting in a uniform slurry. This meticulous process is crucial for maintaining consistency throughout the experiment and enables an accurate assessment of sand retention performance under controlled conditions.

#### 2.3.2 Slurry test

**Fig. 3:**
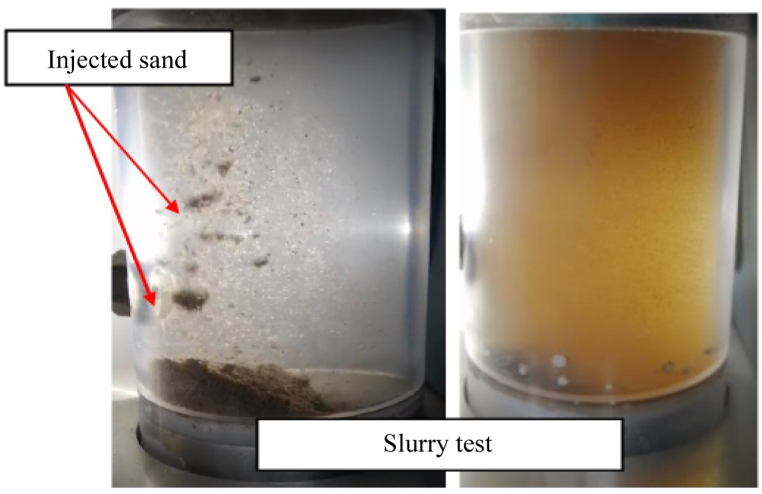
Slurry test of different sand types of reservoir sand retention.

**Fig. 4:**
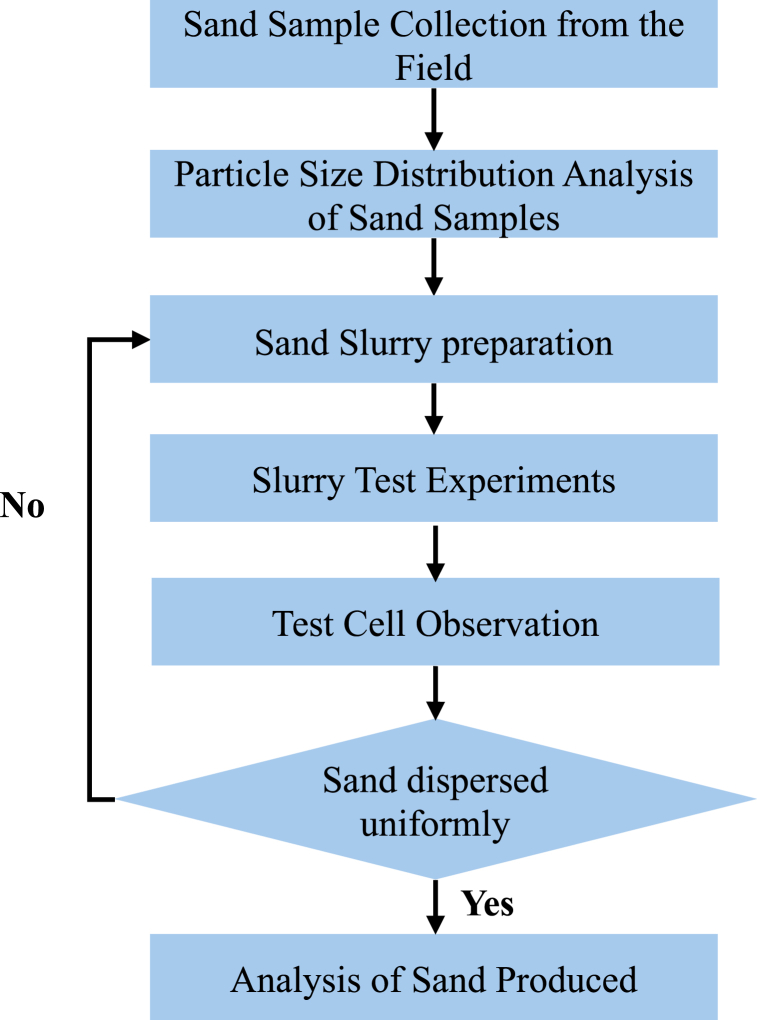
Flow chart of slurry test.

Once the slurry was prepared and placed into the slurry chamber, the test was initiated by setting the fluid flow rate to 140 ml/min and the slurry injection rate to 10 g/min. The pump is activated to fill the sand slurry test chamber and fluid injection line, allowing air to be expelled through the bleed valve. The outlet valve is then opened to allow the slurry to flow One minute after the injection, the sand has been distributed over the screen as shown in Fig. 3. Throughout the process, sand deposition on the screen is carefully monitored. After the sand injection process is completed, the ISCO pumps are stopped, and the produced sand is collected. The measurement of the sand layer on top of the screen is then taken for further analysis. The amount of sand produced, sand retained, and retained permeability were measured and recorded for evaluation [23]. Fig. 4 displays the flow chart of the slurry test being conducted.

#### 2.3.3 Measurement of sand produced

For the measurement of produced sand, the fluid collected during the slurry test is placed in a beaker. Once the injection process is complete, the collected samples are filtered using 11 μm Whatman filter paper to separate the sand. The collected sand samples are then dried in an oven at 70 °C for 6 h to eliminate any residual moisture ensuring precise measurements.

#### 2.3.4 Measurement of sand retained

Measuring retained sand involves determining the distance from the edge of the steel cup to the surface of the retained sand, as viewed through the transparent wall of the sand cell. This method provides a clear and accurate assessment of the sand retention within the system.

#### 2.3.5 Retained permeability

The retained permeability of the screen indicates the degree of plugging of the screen [44]. The initial permeability screen (only) is measured prior slurry test. Following the test, the sand retained is removed, and the screen is measured again to determine the final permeability. The permeability of the sand screen is calculated using the Darcy equation as shown in Eq. (4).

(4) $$K\left(md\right)=\frac{mLQ}{\Delta PA}$$

Where, K = Permeability, μ = Viscosity (cP), L = Length (cm), ΔP = Pressure drop (psi), A = Area (cm^2^). After analyzing the experimental result, the selected samples were further studied through simulation using a mathematical model to better understand the mechanism of the sand retention on the screen.

### 2.4 Mathematical model

#### 2.4.1 Computational Fluid Dynamics (CFD) for liquid flow

CFD is a numerical modelling technique used to simulate various types of fluid flows. The governing equations for CFD are the Navier-Stokes equations which express the transport of three conserved physical quantities, namely Continuity (mass), Momentum and Energy as a set of differential equations as follows [22]:

Continuity Equation:

(5) $$\frac{\partial \rho }{\partial t}+\nabla .\left(\rho \vec{u}\right)=0$$

Where ρ is fluid density, t is time and $\vec{u}$ is flow velocity vector.

Momentum Equation:

(6) $$\rho \frac{\partial \vec{u}}{\partial t}+\left(\rho \vec{u}.\nabla \right)\vec{u}=-\nabla p+\rho b+\nabla .\tau$$

Where p is pressure, b is source term (typically gravity) and τ is viscous stress tensor.

Energy equation:

(7) $$\frac{\partial \left(\rho e\right)}{\partial t}+\nabla .\left(\rho e\vec{u}\right)=\nabla .\left(k\nabla T\right)-p\nabla .\vec{u}+\nabla \vec{u:}\tau +S$$

Where e is internal energy, T is temperature (usually gravity), k is fluid thermal conductivity and $\nabla \vec{u:}\tau$ is double dot product for the irreversible conversion of heat from mechanical energy.

In this study, the Ergun Wen-Yu drag model is being applied in Altair AcuSolve® to calculate the fluid drag force experienced by individual particles. Firstly, the model is selected for its accuracy since it considers both viscous and inertial effects of fluid flow. Besides, the model is very convenient to implement since only a few input parameters are required. The equations of the model are as follows:

(8) $$F_{d}=\beta_{pf}\left(\vec{u}-\vec{v}\right)\rho_{f}$$

where $F_{d}$ is fluid drag force, $\beta_{pf}$ is momentum exchange coefficient, and $\rho_{f}$ is fluid density.

(9) $$\beta_{pf}=150\frac{{\left(1-\varepsilon_{f}\right)}^{2}}{\varepsilon_{f}}\frac{\mu_{f}}{{\left(\phi_{p}d_{p}\right)}^{2}}+1.75\left(1-\varepsilon_{f}\right)\frac{\rho_{f}}{\phi_{p}d_{p}}\left|\vec{u}-\vec{v}\right|\left(\varepsilon_{f}\leq 0.8\right)$$

where $\varepsilon_{f}$ is volumetric fraction by fluid, $\mu_{f}$ is fluid viscosity, $\phi_{p}$ is sphericity and $d_{p}$ is particle diameter.

(10) $$\beta_{pf}=\frac{3}{4}C_{d}\frac{\left|\vec{u}-\vec{v}\right|\rho_{f}\left(1-\varepsilon_{f}\right)}{d_{p}}{\varepsilon_{f}}^{-2.7}\;\left(\varepsilon_{f}>0.8\right)$$

where $C_{d}$ is fluid drag coefficient.

#### 2.4.2 Discrete element method (DEM) for particle motion

**Fig. 5:**
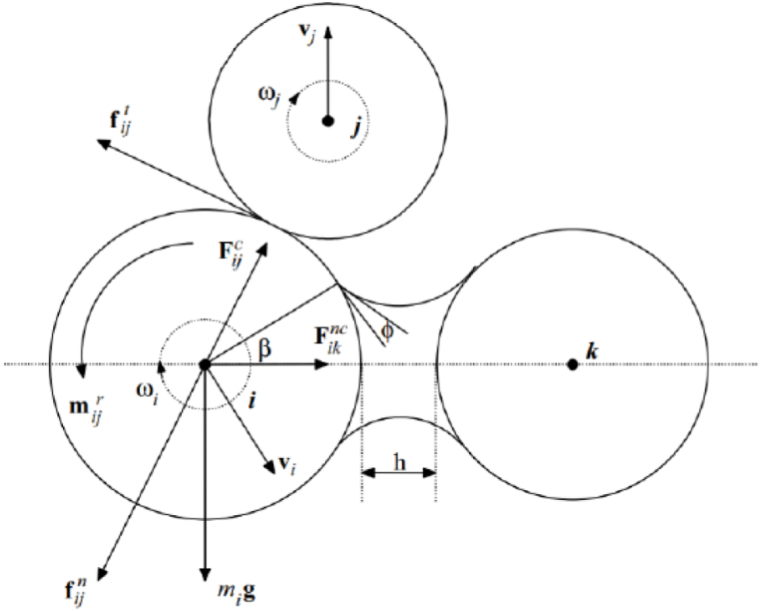
Schematics of the forces exerted by contracting particle *j* and non-contracting particle *k* on particle [23].

DEM is a numerical technique used to simulate the bulk behavior of materials by solving the motion of individual particles using Newton's Second Law of Motion equations. As shown in Fig. 5, the governing equation of DEM consists of both translational and rotational equations of motion as follows:

Translational:

(11) $$m_{i}\frac{d\vec{v_{i}}}{dt}=\sum_{j}F_{ij}^{c}+\sum_{k}F_{ik}^{nc}+F_{i}^{f}+F_{i}^{g}$$

where $m_{i}$ = mass of particle *i*, $\vec{v_{i}}$ = translational velocity of *i*, $F_{ij}^{c}$ = contact force on *i* by *j*, $F_{ik}^{nc}$ = non-contact force on *i* by *k*, $F_{i}^{f}$ = particle interaction force, $F_{i}^{g}$ = gravitational force.

Rotational:

(12) $$I_{i}\frac{d\vec{\omega i}}{\;\text{dt}}=\sum_{i}M_{ij}$$

where $I_{i}$ = moment of inertia of *i*, $\vec{\omega i}$ = rotational velocity of *i*, $M_{ij}$ = torque on *i* by *j*.

For this study, Hertz-Mindlin (no slip) and Standard Rolling Friction are being applied in EDEM to calculate the translational and rotational motion of individual particles respectively. The models are chosen due to their accurate representation which considers elastic deformation and frictional forces as well as their computational efficiency. The equations of the models are as follows in Table 3.where *Fn* = normal force, *E*∗ = equivalent Young's Modulus, *R* = equivalent radius, *δn* = normal overlap, $F_{d\;}^{n}$ = normal damping force, *β* = constant, Sn = normal stiffness, *m* = equivalent mass, $v_{n}^{\vec{\text{rel}}}$ = normal relative velocity, *Ft* = tangential force, *St* = tangential stiffness, *δt* = tangential overlap, $v_{t}^{\vec{\text{rel}}}$ = tangential relative velocity.

**Table 3:** The equation of Hertz-Mindlin (no slip).

**Translation:**	(13) $F_{n}=\left(4/3\right)\;E\;*\;{\sqrt{R}}^{*}\delta_{n}^{1.5}$ (14) $F_{d\;}^{n}{=-2\;\left(5/6\right)\;}^{0.5}\beta \sqrt{S_{n}m}*v_{n}^{\vec{\text{rel}}}$
**Rotational:**	(15) Ft = −S_t_δ_t_ (16) $F_{d\;}^{t}{=-2\left(5/6\right)}^{0.5}\;\beta \sqrt{S_{t}m}*v_{t}^{\vec{\text{rel}}}$

Standard Rolling Friction:

(17) $$\tau_{i}=-\mu_{r}F_{n}R_{i}\vec{\omega_{i}}$$

where $\tau_{i}$ = rolling friction, $\mu_{r}$ = rolling friction coefficient, $R_{i}$ = radius, $\omega_{i}$ = angular velocity.

#### 2.4.3 CFD-DEM coupling approach

**Fig. 6:**
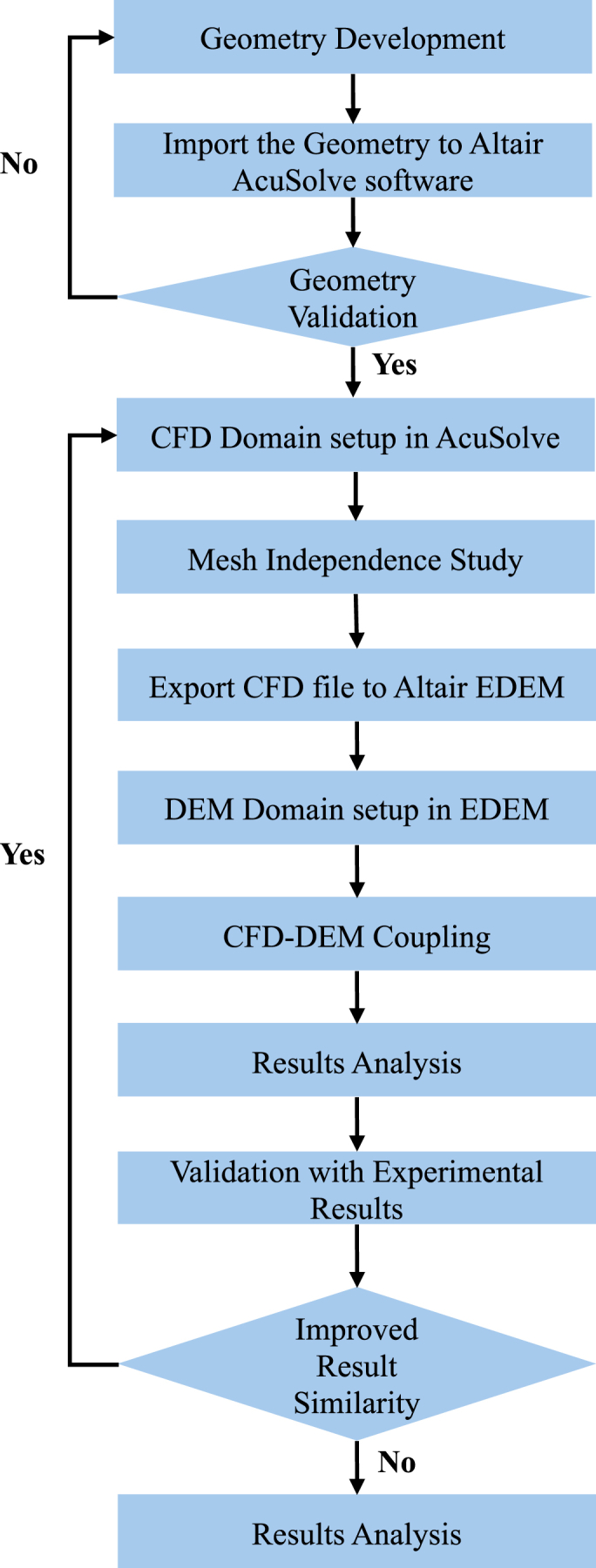
The flow chart of the simulation study.

The Ergun-Wen-Yu drag model was applied in Altair AcuSolve® to compute fluid drag forces exerted on each particle. Meanwhile, in Altair EDEM, the contact models being used are Hertz Mindlin (no-slip) and Standard Rolling Friction for translational and rotational motion of particles respectively. Altair AcuSolve® calculates the drag forces on particles and updates the fluid field based on the particle location and velocity data acquired from Altair EDEM. Then, the location and velocities of each particle will be computed by Altair EDEM using the updated fluid forces data. This computation cycle was looped until the simulation was completed. Fig. 6 displays the flow chart of the simulation study.

### 2.5 Geometry development

**Fig. 7:**
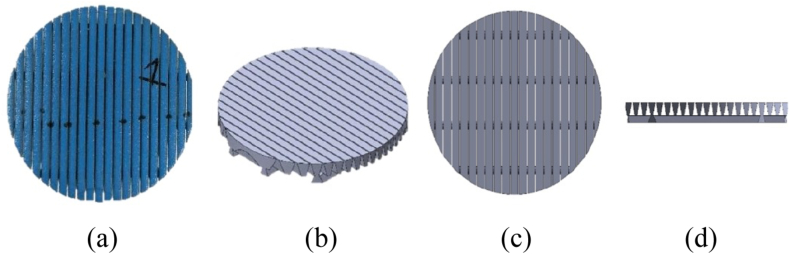
(a) 200 μm WWS coupon used in the experiment, (b) 3D Model developed in isometric view, (c) top view, and (d) side view.

Developing the geometry that closely resembles the actual screen coupons has been the primary focus of this study. The CAD software Autodesk Fusion 360 was used to develop the geometry due to its flexibility and robustness. The 3D model of 200-μm WWS was constructed based on the actual screen used in the sand slurry test. Fig. 7 shows the developed 3D model of WWS.

### 2.6 Simulation conditions for meshing analysis

#### 2.6.1 Mesh independence analysis

**Fig. 8:**
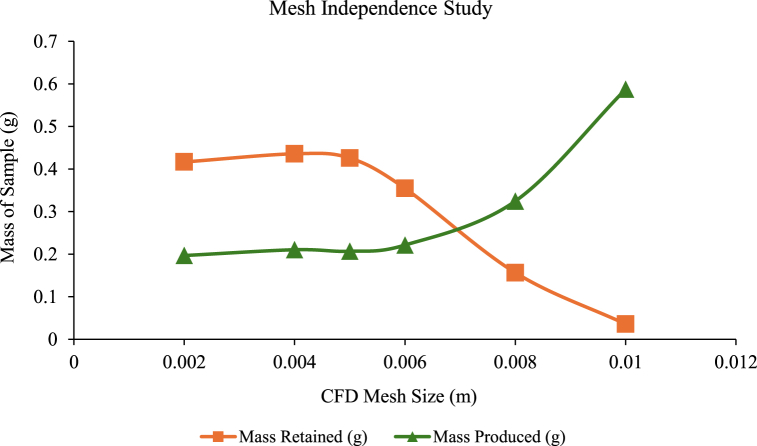
Mesh independence of the proposed wire-wrapped model.

After importing the developed geometry into Altair AcuSolve® and validating it to ensure it is free of any surface or solid defects, a mesh independence analysis was conducted to determine the optimal mesh size for the simulation. The simulation results for WWS were extracted from each trial using different CFD mesh sizes. It was observed that the plot of mass-produced has a high value of 0.5874 g when the CFD mesh size is 0.01m. As the CFD mesh size was reduced, the value gradually decreased and reached a point where the further reduction of mesh size did not cause any significant improvement in the solution accuracy. A similar pattern was noticed for the plot of mass retained, where the value started to increase from 0.03575 g at a mesh size of 0.01 m, reaching a relatively constant value of 0.4 g starting mesh size of 0.005 m. The summary of the results is tabulated in Table 4. From the mesh analysis, it was found that the optimum mesh size is 0.005 m due to the high sand retained and low sand produced. Accordingly, a graph of the mass of the sample retained and produced against the CFD mesh size (in reversed order to observe the trend of result with decreasing mesh size) was plotted as shown in Fig. 8.

**Table 4:**
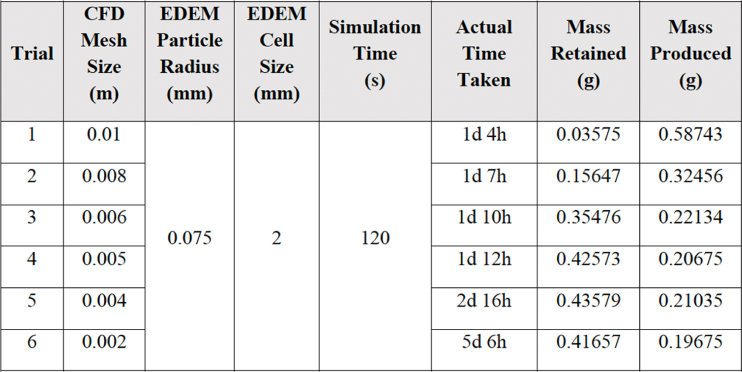
Summary of WWS model simulation result.

This mesh size would then be used in the simulation, where the EDEM cell size will be fixed to 1 mm, to generate a higher number of EDEM cells. This approach captures more detailed information about the particle motion during the EDEM phase. Table 5 represents the CFD- DEM simulation settings for fluid and solid phases used in this study.

**Table 5:** CFD-DEM simulation settings.

Fluid Phase	Solid Phase
**Turbulence Model: Laminar** **Inlet Velocity: 0.15 m/s** **Carrier Fluid Volume Fraction: 1** **Outlet: Pressure Profile** **Walls: No slip (v = 0 m/s)** **Time Step Size: 0.1 s** **Final Time: 120 s** **Multifluid Type: EDEM Bidirectional Material (Water)** **Meshing Size: 0.05**	Particle Diameter: 150 μm (Spherical Shaped) Particle Size Distribution: Lognormal Bulk Material Properties [45] Poisson's Ratio: 0.25 Solids Density: 2500 kg/m^3^ Shear Modulus: 1 × 10^8^ Pa Particle Properties: Mass: 4.41786 × 10^−9^ kg Volume: 1.767158 × 10^−12^ m^3^ Interactions Coefficient [45] Restitution: 0.6 Sliding Friction: 0.5 Rolling Friction: 0.1 Particle Factory Generator Factory Type: Unlimited Number Particle Velocity: 0.15 m/s Generation Rate: 0.025 g/s Simulator Grid: Grid Size: 1 mm

## 3 Results and discussions

### 3.1 Formation criteria

**Fig. 9:**
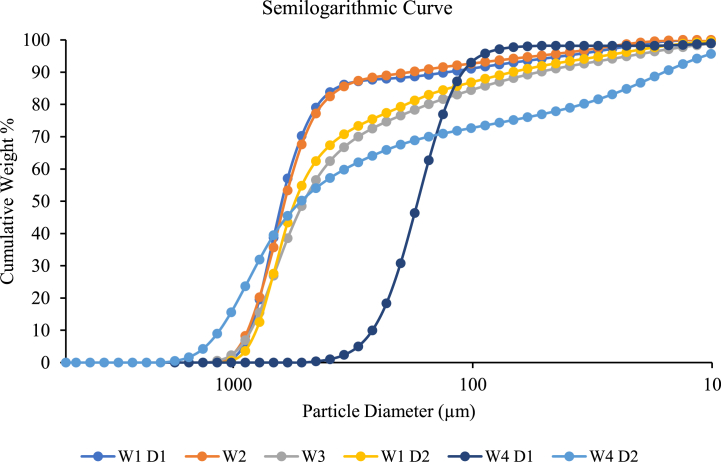
Semilogarithmic graph of sand sample.

Sand particle size distribution (PSD) is crucial for determining the appropriate sizing of filter media in the sand screen technique. The PSD data indicates the particle sizes of the sand formation and the characteristics of the targeted reservoir formation [38,46]. Different reservoirs contain different requirement screen sizing and type. Fig. 9 shows a semilogarithmic graph for the results obtained from the PSD technique. From the graph, the retained percentage has been identified and values such as D10, D40, D50 and D95 were tabulated in Table 6. The details of the Uniformity (UC), Sorting Coefficient (SC) and percentage of fines are also displayed in Table 6. Based on the criteria established by Gillespie et al. [10], all sand samples were classified according to their uniformity and sorting coefficient.

**Table 6:** The particle size distribution and classification of sand samples.

Sand sample	D10	D40	D50	D90	D95	% Fines	UC	Uniformity	SC	Sorting
**W1 D1**	858.04	672.48	625.53	127.89	41.97	5.86	5.26	Non-uniform	20.45	Poorly sorted
**W2**	787.76	521.25	429.73	73.11	32.33	7.17	7.13	Non-uniform	24.37	Poorly sorted
**W3**	849.98	581.21	505.02	53.08	22.37	9.79	10.95	Non-uniform	37.99	Poorly sorted
**W1 D2**	809.24	610.77	584.92	68.45	28.86	8.07	8.92	Non-uniform	28.04	Poorly sorted
**W4 D1**	262.2	184.96	169.68	108.99	92.27	1.82	1.70	Uniform	2.84	Well sorted
**W4 D2**	1143.98	670.75	520.48	15.88	10.78	23.05	42.23	Non-uniform	106.09	Poorly sorted

### 3.2 Experimental screen evaluation

**Fig. 10:**
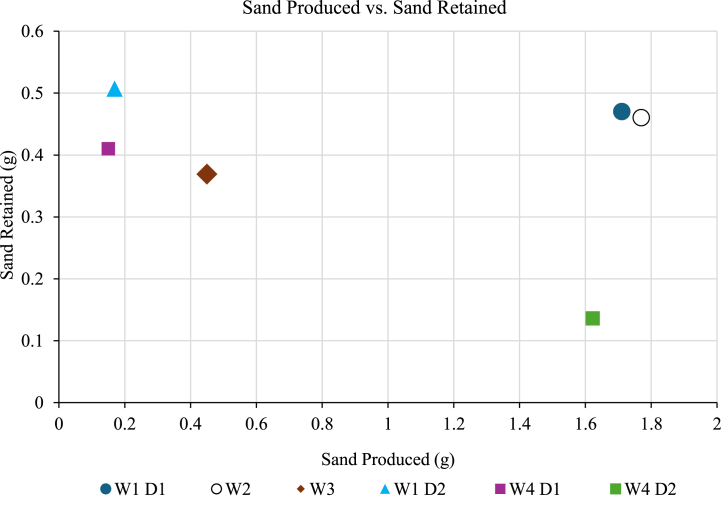
The sand produced, and sand retained of all sand samples.

The sand retained and sand produced is commonly used to evaluate the screen's effectiveness through sand retention tests. Table 7 displays the amount of sand retained, sand produced and retained permeability of different sand samples using a 200-μm wire-wrapped screen. The correlation between sand retained and sand produced was plotted in Fig. 10.

**Table 7:** The performance of wire-wrapped screen in slurry test.

Sand sample	Sand retained (g)	Sand produced (g)	Retained permeability (%)
**W1 D1**	0.47	1.711	34.2
**W2**	0.46	1.77	30.14
**W3**	0.369	0.45	33.17
**W1 D2**	0.507	0.169	50.22
**W4 D1**	0.41	0.15	88.14
**W4 D2**	0.136	1.622	50.22

The W4D1 exhibited the lowest sand production compared to other samples with lower uniformity and poorly sorted sand. Highly uniform and well-sorted sand produces less sand during tests primarily due to its consistent particle size and shape which offers larger void spaces between well-sorted particles [23]. This allows fluid to pass through more easily, which minimizes disturbance to the sand and further reduces sand production. Additionally, the retained permeability was the highest, indicating seamless fluid flow with less plugging effects. The lower amount of sand retained can be attributed to the reduced fines content, which may have dissolved or dispersed more effectively in the solution within the test cell, minimizing accumulation. This finding also suggests that the 200-μm screen size is optimal for mitigating sand production in this specific sand type.

In comparing all sand samples, a graph of sand samples versus sand retained was plotted (Fig. 10). From the graph, it is evident that sample W1D2 shows slightly higher sand production and retention compared to W4D1. Following this, the retained permeability was analyzed for further comparison of screen performance.

**Fig. 11:**
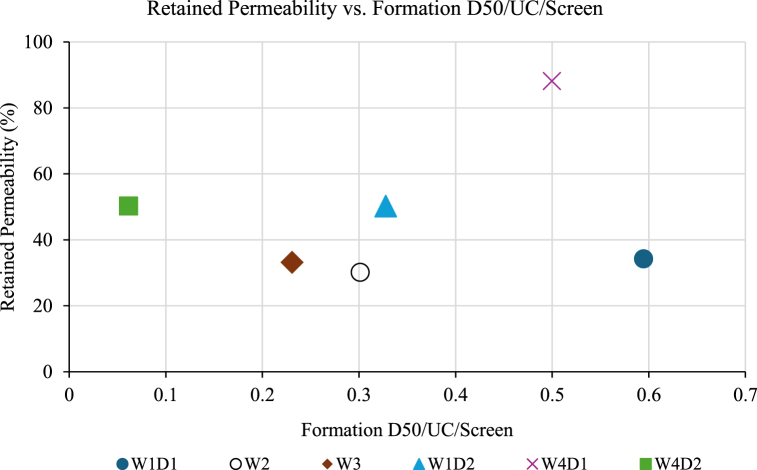
The retained permeability with the ratio of formation D50/UC/Screen performance.

Constien and Skidmore devised a technique for predicting sand control performance through the formulation of a mastercurves graph [47]. This graph is established based on the screen size control device defined as D50/UC/Screen. Performance data of retained permeability against the effective formation size divided by UC and the screen size of the sand control are shown in Fig. 11. Based on the results of retained permeability, sample W4D1 demonstrated retained permeability higher than 50 % when tested with 200-μm WWS followed by W4D2 and W1D2. According to the literature, the acceptable value for retained screen permeability is 50 % or greater [47,48]. Overall, the performance of 200-μm WWS been influenced by the sand characteristics as the highly uniform and well-sorted sand shows the highest retained permeability.

### 3.3 Numerical analysis and model validation

From the experimental analysis, the characteristics of sand samples W1D2 and W4D1 were chosen to study the relation between the fluid and particle flow and understand the effect of some key parameters which may not be straightforward to evaluate experimentally as well as the underlying mechanism behind the occurrences observed in the sand slurry experiment.

CFD approach helps visualize the fluid's movement through the screen's pores and its interaction with the surrounding sand. Meanwhile, DEM is employed to simulate the movement and behavior of individual sand particles. EDEM's built-in mass sensor plays a crucial role in allowing for the measurement of the mass of sand produced and retained, thereby providing precise measurements of these metrics. With the use of DEM, the mass of sand produced and retained is quantified.

**Fig. 12:**
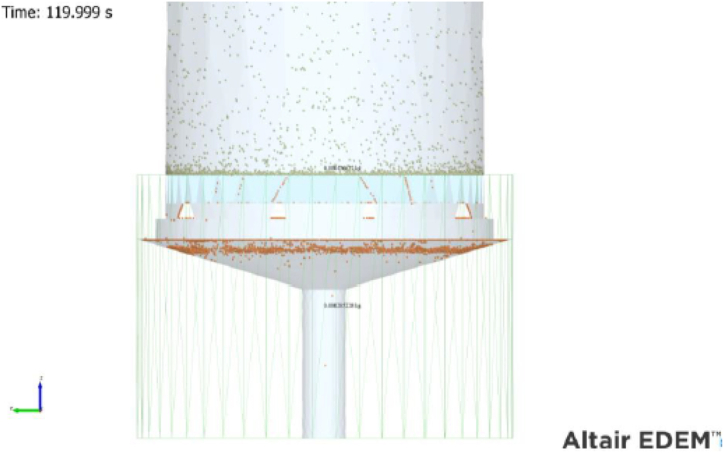
WWS model simulation data extraction for mass sand produced.

As discussed in the previous section, the optimal CFD mesh size of 0.005 m was applied. The model incorporated an EDEM built-in mass sensor positioned at the designated location to quantify the mass of sand passing through the screen, illustrated in Fig. 12.

In Table 8, the simulation outcomes of the WWS model are presented, demonstrating the successful simulation of sand retention using a WWS with 200-μm apertures. The comparison between the simulation results and experimental data was conducted to ascertain the degree of similarity. As mentioned previously, two sand samples which are W1D2 and W4D1 were chosen to compare with the simulation study. The similarities show a high percentage for both samples. Sample W1D2 shows 84.15 % for sand retained and 78.56 % for sand produced while sample W4D1 shows 93.20 % for sand retained and 63.18 % for sand produced respectively. It is noteworthy that both samples exhibit distinct sand characteristics where sample W1D2 is non-uniform and poorly sorted while W4D1 is uniform and well-sorted. This indicates that despite variations in sand composition, the simulation model has been successfully validated and remains reliable in forecasting screen performance and fluid-particle during the sand retention mechanism.

**Table 8:** Comparison of the experimental and simulation results for selected sand samples.

Sand Sample	Produced Sample	Experiment	Simulation	Percentage of Similarity (%)
**W1 D2**	Mass Retained (g)	0.507	0.426622	84.15
	Mass Produced (g)	0.169	0.205228	78.56
**W4 D1**	Mass Retained (g)	0.4	0.426622	93.10
	Mass Produced (g)	0.15	0.205228	63.18

**Fig. 13:**
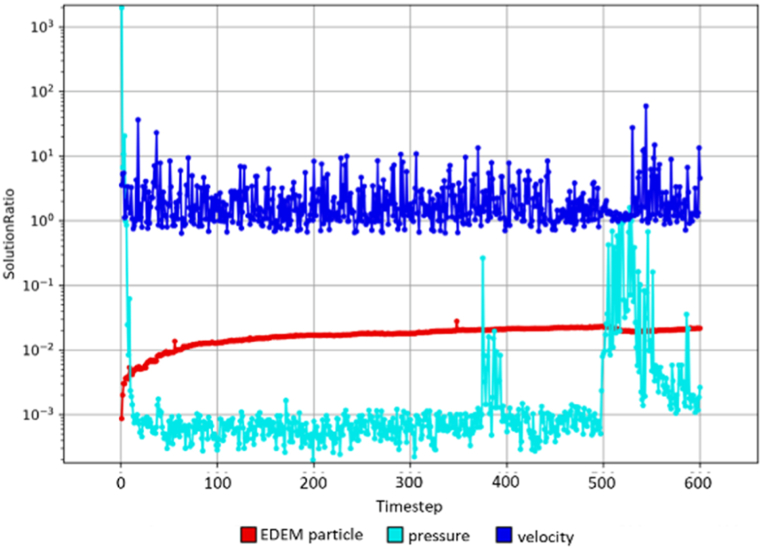
Solution ratio for WWS model.

**Fig. 14:**
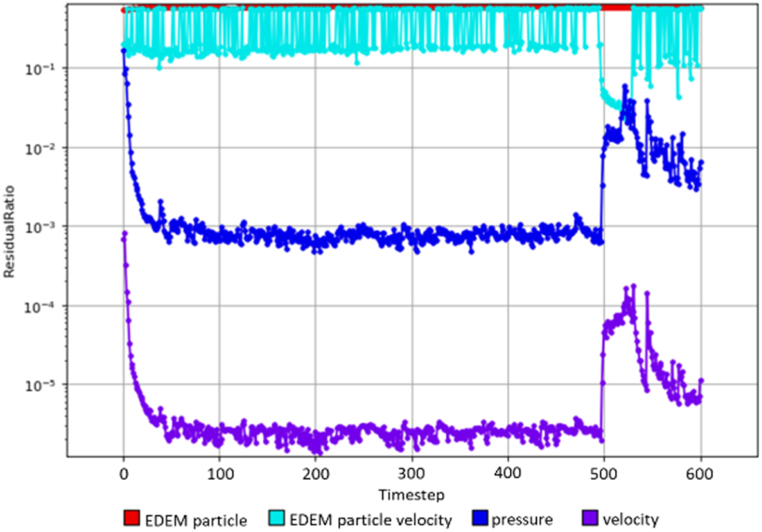
Residual ratio for WWS model.

To further validate the model simulation, Fig. 13, Fig. 14 illustrate the convergence trends for both the solution ratio and residual ratio in this simulation respectively for the WWS model for sample W4D1. The solution ratio graph has reached a steady state indicating that changes in pressure and velocity between consecutive timesteps become very small, meaning that the solution is no longer changing significantly, suggesting that the system is stabilizing. The residual ratio graph shows that the current solution closely matches the governing equations, which means that the simulation error is now minimal and the solution is accurate. According to Lindqvist et al. [49], Altair AcuSolve® only considers a simulation to have reached steady-state convergence when both the solution ratio (the ratio of changes between consecutive timesteps) and the residual ratio (the ratio of the current solution to the governing equations) converge. When both conditions are met, the simulation is considered to have reached a steady state, indicating that the system has stabilized and the results are reliable. Therefore, the simulation study has been validated and progressed to the next stage.

CFD results of this WWS model were worth analyzing to provide insights into the simulation conducted. The results were extracted by slicing the simulation model into symmetrical halves using a plane and generating an interesting contour by applying suitable parameters.

**Fig. 15:**
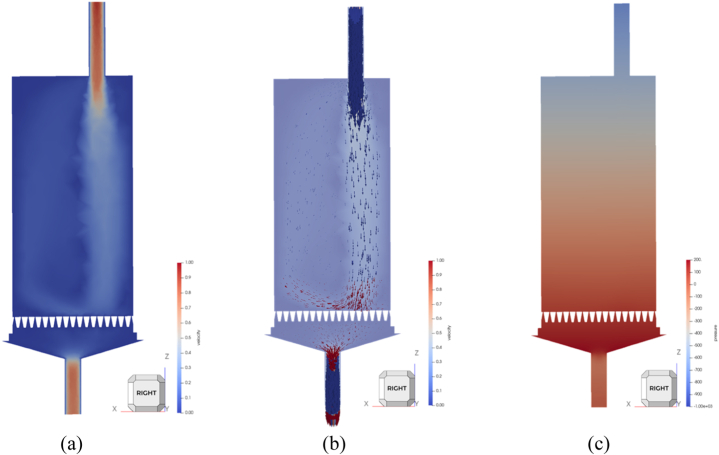
WWS model simulation findings in CFD domain (a) velocity contour, (b) velocity vector, (c) pressure contour.

Fig. 15 illustrates the velocity contour and velocity vectors and pressure contours in WWS model simulation providing valuable insights into fluid behaviour and particle interactions which are crucial for understanding complex systems in slurry tests. Fig. 15 (a) depicts the velocity contour in the WWS model simulation offering a clear visualization of the flow pattern following slurry injection. It could be observed that the fluid flows with higher velocity at the section of inlet and outlet tubes indicated by red contour at these regions. Meanwhile, most of the regions in the main body demonstrated a lower fluid flow velocity as suggested by the blue color at this region. This can be attributed to in order to maintain the constant flow rate, the fluid needed to flow through a narrower passage (inlet and outlet tube) within less time and flow slower through a wider passage (main body).

This information is crucial for understanding how fluids interact with the sand screen and offering insights into the flow dynamics of the slurry flow. In addition, one noticeable phenomenon in these results was the backflow of fluid when reaching the sand screen. The direction of this fluid flow could be observed more clearly from the velocity vector in Fig. 15 (b). It was deduced that the limited passing area on the screen surface has limited the amount of fluid flowing through, thus resulting in the reversed flow of the fluid due to the conservation of momentum.

Fig. 15 (b) represents the velocity vector of the particle motion after the sand was injected. In this simulation study, analyzing the velocity vectors helps to study the particle movement within the fluid which is critical for understanding slurry transport. The maximum velocity is observed at 0.28 m/s at regions of the inlet and outlet tube, whereas the minimum fluid velocity recorded is 0 m/s. The velocity of the fluid flow stream that is reversed after approaching the screen is about 0.15 m/s which is the fluid inlet velocity being inserted during the simulation setup. The fluid that flowed through the screen again sped up when exiting the narrow outlet tube. Validating laminar test calculations is a recognized and accepted criterion to ensure the maintenance of flow and sand retention [50,51]. To determine the flow type, the fluid velocity at the inlet and outlet tube was analyzed and the Reynolds number was calculated (Eq. (18)). Laminar flow provides a controlled environment where the fluid moves smoothly and steadily, reducing the likelihood of disturbances such as turbulence flow that could affect sand production. This ensures that the results reflect the actual sand retention capabilities of the screen under stable, real-world flow conditions.

(18) $$Re=\frac{\rho VD}{\mu }=\frac{\left(1000\right)\left(0.28\right)\left(4.45\times 10^{-3}\right)}{0.001}=1246\;\left(laminar\right)$$

Where Re = Reynolds number (dimensionless), ρ = fluid density (kg/m³), *v* = flow velocity (m/s), *d* = characteristic length or hydraulic diameter (m), μ = dynamic viscosity of the fluid (Pa·s or kg/(m·s)

Last but not least, the pressure contour was as shown in Fig. 15 (c) which indicated that the upper section of the test cell had lower fluid pressure than the lower section of the test cell. For the upper section, the low fluid pressure could be explained by accelerating the fluid flow through the narrow inlet tube, which will result in the decrease of fluid pressure according to Bernoulli's principle. This principle states that in a streamline flow, an increase in fluid velocity occurs simultaneously with a decrease in pressure [52]. At the outlet tube, the fluid pressure is higher because of the deceleration of fluid flow as it exits the system. In this case, as the fluid moves from the main body of the test cell into the outlet tube, it likely encounters resistance or a change in flow velocity, leading to a build-up of pressure at the outlet. Additionally, if there are any backpressure effects or obstructions at the outlet, these could contribute to the increased pressure observed in that region. This is a typical phenomenon in fluid dynamics where pressure rises at exit points due to flow constriction or resistance might be due to the sand screen.

## 4 Conclusion

In this study, a comprehensive slurry test was successfully conducted, focusing on the evaluation of wire-wrapped screens (WWS) with various sand samples. The performance metrics, including sand retention, sand production, and retained permeability, were systematically analyzed. A precise geometry of the WWS was developed and integrated into a coupled CFD-DEM simulation. The results demonstrated a high degree of correlation (93.15 %) between the experimental and simulation outcomes for sand retention, thereby validating the proposed full-geometry CFD-DEM model. This model was further verified using solution and residual convergence graphs, providing insights into fluid flow dynamics through velocity contours, velocity vectors, and pressure contours. These findings offer a deeper understanding of critical factors, such as fluid flow dynamics, particle behavior, and the interactions between the formation and the sand control mechanism. The successful fabrication of a full-scale design model highlights the practical applicability of this approach, making it a valuable tool for future investigations in sand retention testing.

## CRediT authorship contribution statement

**Noorhaslin Che Su:** Writing – review & editing, Investigation, Formal analysis. **Sofyah Anis Izwani Jusof:** Writing – original draft, Software, Methodology, Data curation. **Aimi Zahraa Zainal:** Software, Data curation. **Yeong Yuan Jian:** Writing – original draft, Investigation, Data curation. **Chan Kai Xian:** Writing – original draft, Investigation, Data curation. **Afiq Mohd Laziz:** Validation, Supervision, Software. **Muhmmad Fadhli Muhammad:** Supervision, Project administration, Funding acquisition. **Javed Akhbar Khan:** Writing – review & editing, Validation. **Abdul Hazim Abdullah:** Writing – review & editing, Validation. **Mohd Azuwan Maoinser:** Supervision, Funding acquisition, Conceptualization.

## Data Availability statement

The data that support the findings of this study are available from the corresponding author upon reasonable request.

## Declaration of competing interest

The authors declare that they have no known competing financial interests or personal relationships that could have appeared to influence the work reported in this paper.
